# Cellulose–Hemicellulose–Lignin Interaction in the Secondary Cell Wall of Coconut Endocarp

**DOI:** 10.3390/biomimetics8020188

**Published:** 2023-05-04

**Authors:** Sharmi Mazumder, Ning Zhang

**Affiliations:** Department of Mechanical Engineering, Baylor University, Waco, TX 76706, USA

**Keywords:** coconut endocarp, cellulose, hemicellulose, lignin, nanoscale deformation mechanisms

## Abstract

The coconut shell consists of three distinct layers: the skin-like outermost exocarp, the thick fibrous mesocarp, and the hard and tough inner endocarp. In this work, we focused on the endocarp because it features a unique combination of superior properties, including low weight, high strength, high hardness, and high toughness. These properties are usually mutually exclusive in synthesized composites. The microstructures of the secondary cell wall of the endocarp at the nanoscale, in which cellulose microfibrils are surrounded by hemicellulose and lignin, were generated. All-atom molecular dynamics simulations with PCFF force field were conducted to investigate the deformation and failure mechanisms under uniaxial shear and tension. Steered molecular dynamics simulations were carried out to study the interaction between different types of polymer chains. The results demonstrated that cellulose–hemicellulose and cellulose–lignin exhibit the strongest and weakest interactions, respectively. This conclusion was further validated against the DFT calculations. Additionally, through shear simulations of sandwiched polymer models, it was found that cellulose–hemicellulose-cellulose exhibits the highest strength and toughness, while cellulose–lignin-cellulose shows the lowest strength and toughness among all tested cases. This conclusion was further confirmed by uniaxial tension simulations of sandwiched polymer models. It was revealed that hydrogen bonds formed between the polymer chains are responsible for the observed strengthening and toughening behaviors. Additionally, it was interesting to note that failure mode under tension varies with the density of amorphous polymers located between cellulose bundles. The failure mode of multilayer polymer models under tension was also investigated. The findings of this work could potentially provide guidelines for the design of coconut-inspired lightweight cellular materials.

## 1. Introduction

The biological structural materials [1,2,3,4] have aroused the great interest of materials scientists and engineers because of their great potential in stimulating the development of novel biomimetic materials. Coconut shell is one of the bio-polymer-based cellular materials. It has three distinct layers: the outer lethargy brown exocarp, the middle thick fibrous mesocarp, and the innermost hard and tough woody endocarp. Herein, we mainly focused on the endocarp part because it features a combination of high hardness, high stiffness, high strength, high impact resistance, and lightweight, which are normally mutually exclusive in synthesized man-made materials [5].

**Figure 1:**
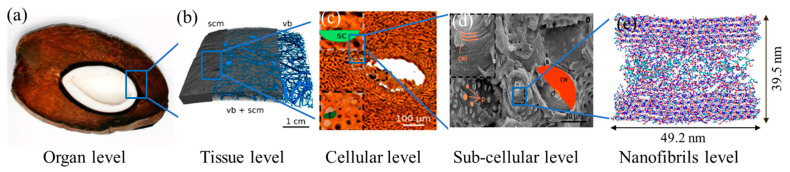
The hierarchical structure of coconut endocarp [6]: (**a**) a broken coconut, (**b**) scleroid matrix and vascular bundles, (**c**) scleroids, (**d**) cell wall layers, and (**e**) nanofibrils of the secondary cell wall.

Experimental and computational studies have been conducted to understand the functional morphology of the endocarp. Similar to most biomaterials, the endocarp exhibits a complex multi-scale hierarchical structure (Figure 1). Four different structural organizations of tissues, cells, subcellular (cell wall layer), and nanofibrils were identified in the endocarp at different length scales [6,7]. At the macroscale, a porous network was observed with vascular bundles resembling hollow channels interspersed in the dense scleroid matrix that runs through the center of the shell (Figure 1b). Channels network runs in all directions whereas mainly the larger channels run parallel to the shell surface. At the microscale, the matrix is composed of thick-walled sclereid cells and tracheid (Figure 1c). The sclereid cells are graded as a cellular structure having walls of 7 ± 2 µm in thickness, which occupies 89.2% of the cross-sectional area. These cell walls consist of several layers, i.e., secondary cell walls, which are traversed by pit canals and porosity of approximately 1 µm (Figure 1d). At the nanoscale, each layer of the secondary cell wall is further composed of crystalline cellulose microfibrils that are embedded in the soft amorphous matrix of hemicellulose, lignin and a minor amount of pectin and proteins. In an experimental study of bamboo, the hemicellulose and lignin matrix were found not only to facilitate the load transfer but also to adhere cellulose microfibrils together to achieve extraordinary strengthening behavior [8].

On the other hand, the mechanical properties of endocarp do not appear to be compromised by its high porosity. Coconut endocarp has a compressive strength of 230–270 MPa [9,10], comparable to mild steel (~250 MPa) [5,11]. Its work of fracture (24.8 ± 8.4 kJ/m^2^) [9] is orders of magnitude higher than that of most man-made porous/cellular materials, such as porous ceramics (12.0–16.0 J/m^2^) [12,13]. Coconut endocarp is also renowned for its high hardness (Vickers hardness: 500–540 MPa) [9] and stiffness (8.0–10.0 GPa) [10]. Coconut endocarp also exhibits outstanding properties in comparison to other natural cellular materials, such as wood. In addition, the mechanical properties of endocarp are improved with the age and particular orientations, hence becoming more anisotropic [7]. In the latitude direction, old coconut endocarp exhibits great damage tolerant properties, such as 82% higher strength and >50% higher crack growth toughness than those of the younger endocarp [14].

Motivated by the superior mechanical performance of coconut endocarp, great efforts have been dedicated to revealing the underlying plastic deformation mechanisms. Most of the early investigations of coconut endocarp were conducted by the microscale experimental approaches. To date, the proposed toughening mechanisms have primarily focused on the role of the microstructure of cell wall layers, such as the surface pits deflect the crack propagation path. Additionally, old coconut shell possesses denser structure by developing open channels, which further confine the crack and consequently increases the fracture toughness [7]. The enhanced hardness of old endocarp was assumed to be due to the thickening and lignification of cell wall as aging evolves. High strength and stiffness were attributed to the dense structure of old coconut because it provides uniform load distribution. Several numerical models, such as the finite element method [15], were developed to simulate the microstructure at mesoscale with an attempt to predict the mechanical characteristic of wood. However, those studies can only explain the mechanical response from the geometry of the structure rather than from the viewpoint of materials. As for coconut shell, the numerical study was limited on the fiber arrangement of mesocarp [16].

Despite of the achievement obtained, a quantitative understanding of the mechanisms by which the coconut endocarp accomplishes its extraordinary mechanical function, particularly the fundamental information at nanoscale, remains missing. Considering the challenges and limitations of experiments at fine scale, atomistic modeling is an alternative method to establish an underlying interpretation of how the complex architecture/structure of the coconut endocarp governs its extraordinary properties. Molecular dynamics (MD) method has been successfully employed to study the failure mechanisms of wood at nanoscale [3,4]. However, no atomistic studies of coconut endocarp have been reported so far. To develop new biomimetic multifunctional materials, the underlying governing mechanisms and design principles of the endocarp are needed. It is worth noting that although wood and hard endocarp share similar components, they exhibit different compositions and structures. Wood is a cellulose dominated material with ~40–50% cellulose, 10–40% hemicellulose, and 17–25% lignin [17]. In contrast, the cell wall of endocarp is more lignified with ~29% cellulose, ~20–30% hemicellulose, and ~44% lignin [17,18,19].

Given the importance of the interaction between different types of polymers in the overall mechanical performance of endocarp, improving our understanding of the atomic processes of deformation will be a prerequisite and crucial task in material science. To this end, in this study, we employed the classic MD simulation method to identify the interactions of three main components of coconut endocarp, i.e., cellulose, hemicellulose, and lignin, at nanoscale, and investigate the mechanical properties and corresponding failure mechanisms. The paper is organized as follows: Section 2 briefly discussed the computational method and models. In Section 3, we presented the simulation results and discussion. The conclusions were summarized in Section 4.

## 2. Materials and Methods

### 2.1. Basic Polymer Units

**Figure 2:**
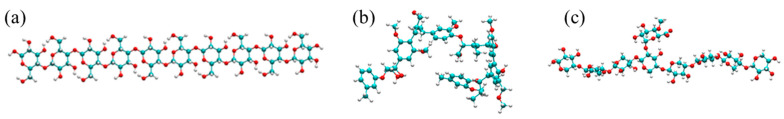
Atomic configurations of (**a**) cellulose; (**b**) lignin; and (**c**) hemicellulose. The blue, red, and white atoms represent carbon, oxygen, and hydrogen, respectively.

Cellulose, lignin, and hemicellulose (Figure 2a–c) are the key polymers of the coconut endocarp. Cellulose and hemicellulose both are carbohydrates, while lignin is crosslinking heterogeneous polymer made by phenolic precursors.

Cellulose (C_6_H_10_O_5_) is the most plentiful and beneficial natural bio-polymer [20]. It is the predominant component of the secondary cell wall of wood. X-ray and neutron diffraction experiments [21,22] revealed that micro fibrils of cellulose Iβ are crystalline structured, and they are homo polysaccharide linear chain consists of covalently connected β-1,4-glycosidic linkages of hundreds to thousands. It was reported that each cellulose bundle consists of 5 to 48 microfibril chains [23]. The unit cell of cellulose is monoclinic (P2_1_) with lattice parameters of *a* = 7.78 Å, *b* = 8.20 Å, *c* = 10.38 Å, *α* = *β* = 90°, *γ* = 96.5° [3,24]. Cellulose bundles perform a significant role in reinforcing the plant cell wall attributing to intermolecular hydrogen bonding network between adjacent chains [21]. In the current study, the cellulose bundle consists of five layers of polymers, and each layer contains six chains, and each chain consists of 24 D-glucose residues.

Hemicellulose is a diverse group of polysaccharides [25]. Unlike cellulose, hemicellulose exhibits a random and amorphous structure. It is composed of short molecules, leading to a lower degree of polymerization. Cellulose microfibrils are surrounded by hemicellulose and lignin. In coconut endocarp, xylans [26], which consists of a linear *β*-1, 4-linked D- xylopyranose (Xylp) backbone with *α*-D-glucuronic acid (GlcA), is taken into consideration.

Lignin is the second most abundant class of biopolymers on Earth [27]. Among the three key polymers, lignin performs a vital role in the formation and mechanical performance of the coconut endocarp. It features a large group of aromatic biopolymers, which has highly branched chemical structure with different functional groups, such as methoxyl (CH_3_O), carboxyl (COOH), and carbonyl (C=O) [28,29]. Lignin also exhibits an amorphous morphology, and it crosslinks hemicellulose and cellulose microfibrils. The most common linkages identified in these polymers are β-O-4, α-O-4, β-5, β–β, 5-5’, 4-O-5, and β-1’. In this study, simplified lignin structure is made mainly consists of syringyl unit with β-O-4 linkages.

### 2.2. Simulation Methods and Procedure

**Figure 3:**
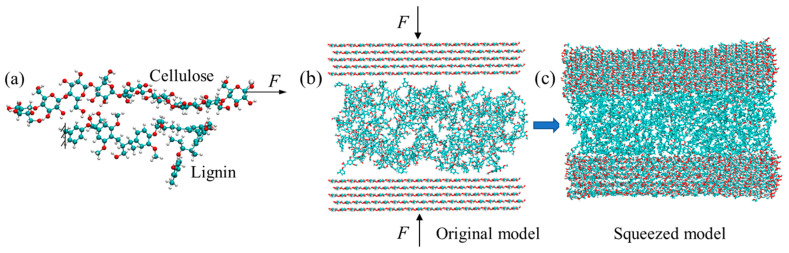
(**a**) A representative of SMD model; (**b**) original; and (**c**) squeezed sandwiched polymer models under uniaxial compressive loading of *F*.

To reveal the interfacial splitting mechanisms among three different polymers, steered molecular dynamics (SMD) technique was employed to mimic the Atomic Force Microscopy (AFM) experiment. SMD method has been used to trace the conformational changes and to determine the interaction between protein chains [1]. In the endocarp, it is still unclear of the arrangement of polymers. To this end, three pairs of polymer couples, i.e., cellulose–hemicellulose, cellulose–lignin, and hemicellulose–lignin, were generated, as represented in Figure 3a. In each pair, one end of a polymer was fixed to prevent movement with the loading, a constant velocity was applied on the free end of the other polymer to simulate shear between them. Before pulling, the system was fully relaxed to ensure to reach the energy equilibrium.

To validate the SMD results, we further performed the DFT calculations. Periodic boundary conditions were applied on the three polymer couples. The exchange correlation energy of interacting electrons was treated by both the Perdew–Burke–Ernzerhof (PBE) version of the generalized gradient approximation (GGA) [30]. All calculations were carried out using the Material Studio. Through calculating the adsorption energies between adjacent polymer chains after full geometry optimization, the chemical bond strength can be quantified. The adsorption energy is defined as:

(1) $$E_{ads}=-\left[E_{system}-\left(E_{adsorbent}+E_{adsorbate}\right)\right]$$

Classic MD tension simulations were carried out to study the mechanical properties of the sandwiched polymer systems (Figure 3b,c). The model is composed of two cellulose microfibrils bundles at the top and bottom, with each bundle consists of five layers of polymer chains. Pure hemicellulose/lignin or the combination of them are embedded in-between the cellulose bundles. The PACKMOL [31] was utilized to construct the models (Figure 3b), which have a dimension of 38.5 nm × 32.8 nm × 49.2 nm with a total number of atoms around 30,000. Since hemicellulose and lignin possess amorphous morphologies, it is challenging to create a model that ensuring a close contact between polymers, particularly between crystalline cellulose bundles and amorphous hemicellulose/lignin. Our strategy was to apply compressive loading that perpendicular to the cellulose bundle to eliminate the spacing (Figure 3c). Afterward, the entire system was allowed to carry out full relaxation for a period of 1 ns using Nose-Hoover Thermostat (NVT) ensemble at room temperature (298 K). Periodic boundaries were applied along the direction of cellulose microfibril bundles. Shake algorithm was applied to all bonds containing hydrogens. The Ewald summation [32] method with an accuracy of 1 × 10^−4^ kcal/mol was used for the long-range columbic interactions.

Shear and tension simulations of the sandwiched polymer models were carried out. Shear and tensile loadings with strain of 8.0 × 10^7^/s, which has been proved to be slow enough to avoid numerical effect [33], were applied on the top and bottom cellulose bundles to test the mechanical response. Free surface boundary condition was applied to ensure shearing and stretching without any truncation. The modulus of toughness, or strain energy density, was calculated by integrating each stress–strain curve over all the applied strain, to equivalently estimate the toughness of different composite models.

For biopolymers, the potentials of AMBER [34,35], CHARMM [36,37,38], GROMACS [39], and Polymer Consistent Force-Field (PCFF) [40] are well known to simulate structural deformations. In the current work, SMD simulations were performed using the AMBER Package with the GLYCAM_06j force field [41]. First principle studies based on density functional theory (DFT) were conducted using the Biovia Material Studio to validate the results of SMD simulations. For classic MD simulations, LAMMPS simulation package [42] with PCFF potential was employed.

## 3. Results and Discussion

### 3.1. SMD Shear Simulations of Polymer Couples

**Figure 4:**
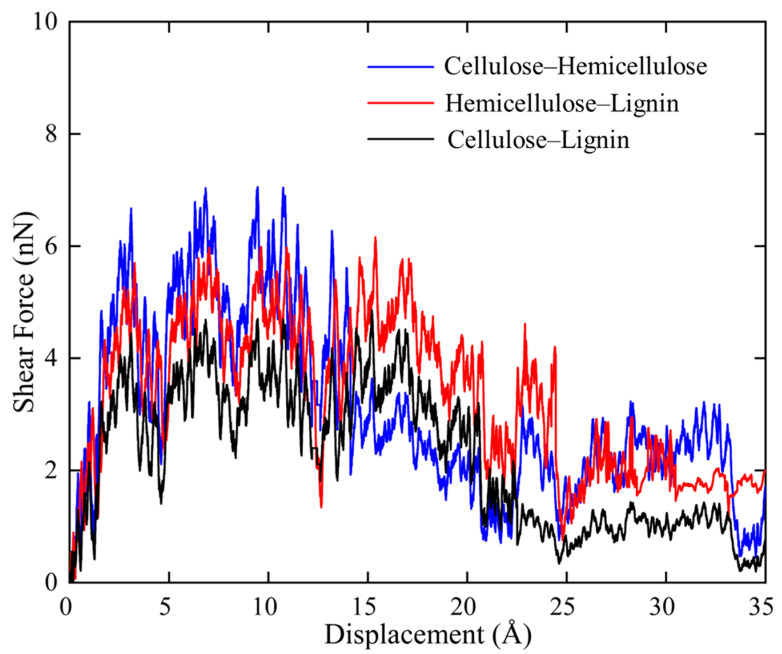
Force–displacement curves measured during the SMD shear simulations.

In SMD shear simulations, polymer chains in each couple were placed parallel to each other with an optimum distance so that they can easily interact during stretching. Force-displacement (f-d) curves of the three tested couples, i.e., cellulose–hemicellulose, cellulose–lignin, and hemicellulose–lignin, were then plotted and compared in Figure 4. It is noted that all three curves show a quite serrate feature. By tracking the atomic trajectories, it is found that H-bonds are formed between the hydrogen atoms and hydroxyl group of the adjacent polymer chains. As one polymer chain is under pulling, H-bonds are first be stretched, which leads to local increases of the force. Then, the stretched H-bonds lose their ability to resist further loading and followed by instantaneous collapse, which is reflected as sudden force drops on the f-d curves. Thereafter, new H-bonds form, are stretched, and finally, break. Such circle of H-bonds reforming and breaking repeats during the overall shear process and it can be used to explain the local fluctuations in f-d curves in Figure 4.

On the other hand, the three polymer bundles exhibit distinct maximum shear force. Cellulose–hemicellulose shows the maximum value of 7.0 nN, while cellulose–lignin shows the minimum value of 4.5 nN. This phenomenon indicates strong interaction between cellulose–hemicellulose and a weak interaction between cellulose–lignin. Considering the formation of H-bonds requires oxygen as the acceptor, the amount of oxygen should play a decisive role in the H-bonds formation. The density of oxygen for cellulose, hemicellulose and lignin are 27.6%, 24.2%, and 10.5%, respectively. Accordingly, more H-bonds form between cellulose–hemicellulose during shear, which consequently leads to strong interface. Since the real morphology of endocarp is not available so far, we hypothesize based on our SMD simulation results that cellulose bundles are surrounded by hemicellulose, then they overall are embedded in a lignin matrix.

**Figure 5:**
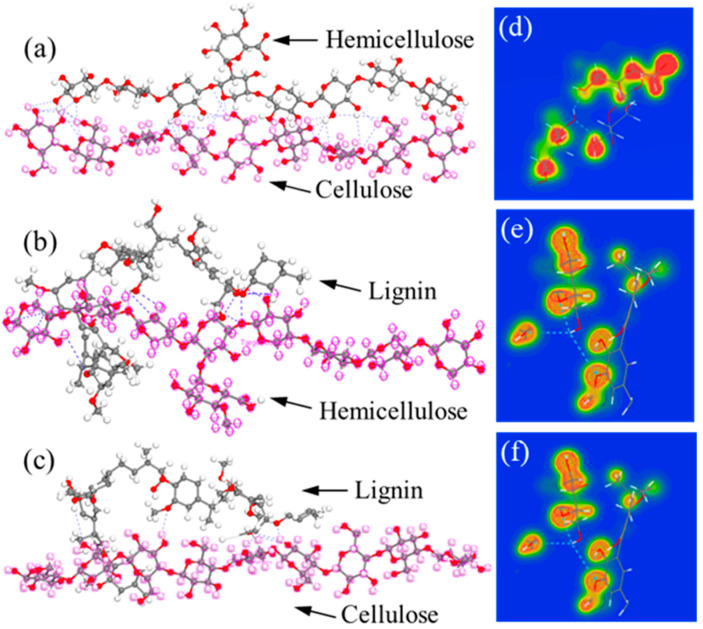
Adsorption sites and H-bonds formed between (**a**) cellulose–hemicellulose, (**b**) hemicellulose–lignin, and (**c**) cellulose–lignin. The electron CDD of (**d**) cellulose–hemicellulose, (**e**) hemicellulose–lignin, and (**f**) cellulose–lignin couples.

**Figure 6:**
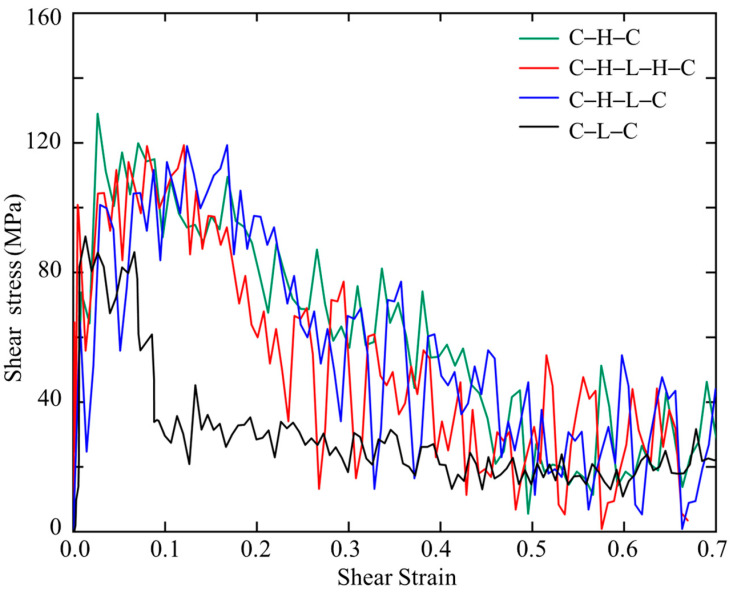
Stress–strain curves of sandwiched polymers under shear.

By DFT calculations, the obtained adsorption energies of cellulose–hemicellulose, hemicellulose–lignin, and cellulose–lignin couples are −135.1 kcal/mol, −106.765 kcal/mol, and −87.653 kcal/mol, respectively, which is consistent with the rank of the calculated maximum shear force from SMD simulations. To quantify the H-bonds formed between polymer chains, we present the configurations with the most stable adsorption sites in Figure 5a–c. It can be seen that cellulose–hemicellulose and cellulose–lignin show the maximum and minimum number of H-bonds, respectively. To gain a direct observation of the chemical bonding, we further analyze electron charge density distribution (CDD) [43], as shown in Figure 6d–f. Red area around the elements indicates the high electron density, while blue area represents low electron density. It can be noted that the cellulose–hemicellulose bundle exhibits the highest electron density, which implies the strongest interaction between them.

### 3.2. Shear Simulations of Sandwiched Polymers

In this section, four sandwiched polymer models, i.e., cellulose–hemicellulose–cellulose (C–H–C), cellulose–hemicellulose–lignincellulose–lignin–hemicellulose–cellulose (C–H–L–H–C), cellulose–hemicellulose–lignin–cellulose (C–H–L–C), and cellulose–lignin–cellulose (C–L–C), were constructed. Shear loading was applied on the top and bottom cellulose bundles to test the mechanical response of endocarp under shear.

Figure 6 shows the stress–strain relations of the sandwiched polymer composites under shear. It is noted that the C–H–C, C–H–L–H–C, and C–H–L–C models show similar stress–strain patterns that after an abrupt increase, the stress reaches a plateau, then followed by gradual decrease until approaches a steady state. In contrast, C–L–C exhibits a shorter elastic deformation stage and a significant narrower stress plateau region in the plastic flow region. Such differences lead to different areas beneath the stress–strain curves, i.e., the modulus of toughness. Additionally, among the four sandwiched structures, C–H–C and C–L–C show the highest and lowest shear strengths of 137 MPa and 90 MPa, respectively. Therefore, we conclude that C–H–C is the strongest and toughest model, while C–L–C is the weakest and most brittle model, which is consistent with the SMD results.

**Figure 7:**
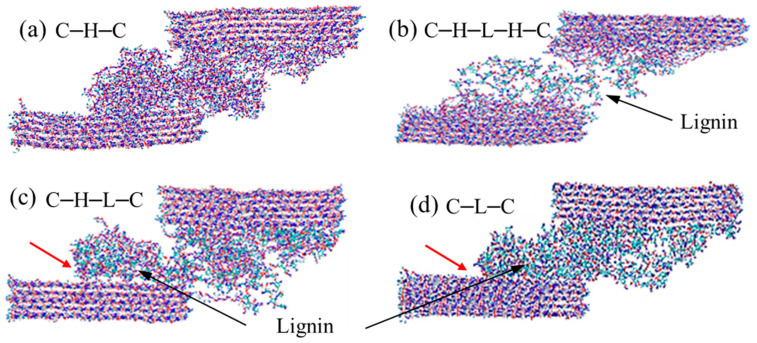
Snapshots of (**a**) cellulose–hemicellulose–cellulose (C–H–C), (**b**) cellulose–hemicellulose–lignincellulose–lignin–hemicellulose–cellulose (C–H–L–H–C), (**c**) cellulose–hemicellulose–lignin–cellulose (C–H–L–C) and (**d**) cellulose–lignin–cellulose (C–L–C) under shear. Red arrows denote the peeling off interfaces between cellulose–lignin.

To gain insights into the failure mechanisms behind the stress–strain curves, we tracked the deformation trajectories and presented the snapshots of the four configurations at the strain of 0.4 in Figure 7. It can be seen from Figure 7a that, during the shear of C–H–C model, cellulose and hemicellulose are intimately connected on their interfaces, resulting in an entangled hemicellulose lump rotating, and moving with the cellulose bundle. When lignin is sandwiched by cellulose–hemicellulose (Figure 7b), one can note that hemicellulose is prone to stick to the cellulose surface, and the fracture eventually occurs in the middle lignin region, which again demonstrates the strong interaction between cellulose–hemicellulose. As for the cases of C–H–L–C and C–L–C, the lignin units are noticed to be easier to peel off the cellulose bundle, leaving clean surfaces behind, as marked by red arrows in Figure 7c,d. Close observation of the deformed configurations manifests that no covalent bonds break during the shear deformation, owing to the ultra-high strength of covalent bonds comparing to other non-bonded interactions, such as H-bonds. It is worth mentioning that for the case of C–H–L–C, as shown in Figure 7c, besides the peel-off of lignin from the cellulose surface, another dominant failure was observed between hemicellulose-cellulose. As aforementioned, the interaction between hemicellulose-cellulose is stronger than that of cellulose–lignin. Therefore, slight differences are observed in the cases of C–H–L–H–C and C–H–L–C. On the other hand, the simulated models in this work are relatively small, hence the effect of different polymer chains on stress–strain curves are not so significant.

### 3.3. Tension Simulations of Sandwiched Multilayer Polymers

**Figure 8:**
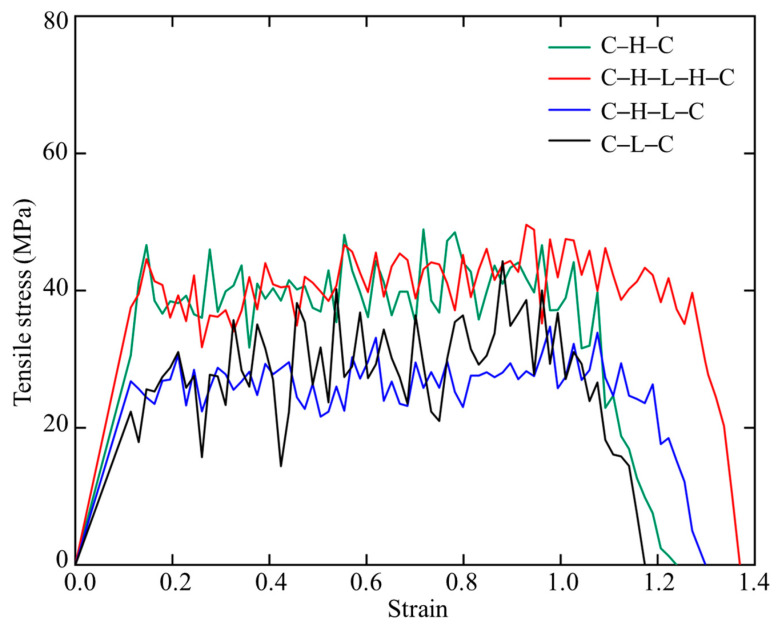
Stress–strain curves of sandwiched polymers with different compositions and arrangements under tension.

Uniaxial tensile loading with same strain rate of 8.0 × 10^7^/s was applied on the four sandwiched polymer models in Section 3.2 to investigate the mechanical response and underlying failure mechanisms. Figure 8 compares the stress–strain curves of the four models under tension. One can note that the models with different arrangements show rather different plastic deformation behaviors although with similar pattern. After a short linear elastic stage, a remarkable stress plateau was observed to dominate the overall deformation, followed by a sudden stress drop. C–H–C and C–H–L–H–C show an average yielding stress of 42 MPa, whereas C–H–L–C and C–L–C exhibit a value as low as 26 MPa. The modulus of toughness of C–H–C and C–H–L–H–C is also much higher than that of the C–H–L–C and C–L–C, which indicates a higher ductility in C–H–C and C–H–L–H–C. The elastic modulus (*E*), i.e., the slope of stress–strain curves in elastic region, varies with the arrangement, and is in the order of *E*_C-H-L-H-C_ > *E*_C-H-C_ > *E*_C-H-L-C_ > *E*_C-L-C_. The fluctuation of the stress–strain curve is due to the disentanglement of amorphous hemicellulose and lignin chains. For the different sandwiched polymer models, it is difficult to quantify the degree of entanglement. Hence, the local stress peaks of C–L–C may be higher than those of the C–H–L–C model.

**Figure 9:**
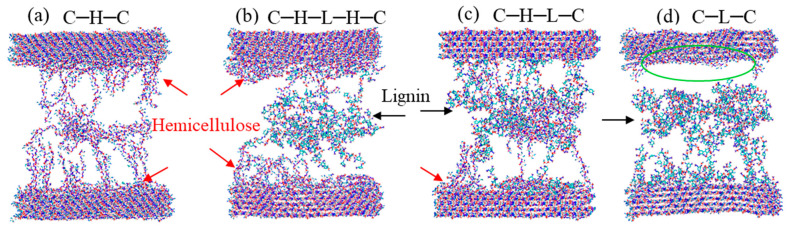
Snapshots of (**a**) cellulose–hemicellulose–cellulose (C–H–C), (**b**) cellulose–hemicellulose–lignincellulose–lignin–hemicellulose–cellulose (C–H–L–H–C), (**c**) cellulose–hemicellulose–lignin–cellulose (C–H–L–C) and (**d**) cellulose–lignin–cellulose (C–L–C) under uniaxial tension. The red and black arrows denote hemicellulose and lignin, respectively. The green ellipse marks the clean rupture interface.

In Figure 9, we presented the snapshots of the configurations under tensile strain of 1.1. It can be seen from Figure 9a that, in the case of C–H–C, hemicellulose chains have a favorable tendency to adhere onto the surfaces of cellulose bundles, as denoted by red arrows. With lignin was added to generate the C–H–L–H–C model, same bonding mode was observed in Figure 9b, i.e., hemicellulose strongly attaches to the cellulose, which thereby leads to a fracture between hemicellulose and lignin. Similar fracture behavior was observed in the case of C–H–L–C, as shown in Figure 9c. It is worth mentioning that, in the original model, hemicellulose and lignin are built layer-by-layer in the order of C–H–L–C. However, after relaxation, they somehow entangled together and shift their positions. Particularly for hemicellulose, which is prone to stick to the cellulose surface, due to the strongest adsorption energy between them. Therefore, one can note from Figure 9c that the hemicellulose chain is attached to the surface of the bottom cellulose bundle after rupture, rather than the original arrangement of lignin and cellulose. On the other hand, unlike crystalline materials, the surfaces of polymers, particularly the in-between amorphous region, are not smooth, which could potentially initiate the stress concentration and lead to crack propagation from the defected sites. Hence, the fracture does not necessarily occur on the interface of C–L.

However, for the case with pure lignin added (Figure 9d), a clean detachment was detected between cellulose and lignin, as marked by green ellipse. Through the comparison of the failure behaviors in Figure 9, we concluded that cellulose–hemicellulose has a strong interaction on the interface, while cellulose–lignin or hemicellulose–lignin is weakly interacted. In addition to the low oxygen density in lignin, the weak interaction can also be explained from the viewpoint of the structural morphology. Lignin is a cross linked polymer; thus, the contact area between cellulose–lignin is less than that between cellulose–hemicellulose, which reduce the probability of H-bonds formation between cellulose–lignin.

Tracking the detailed deformation process revealed that disentanglement of amorphous polymer chains dominates the early deformation, i.e., elastic deformation. Thereafter, the backbones of the polymer chains are stretched to straight shape. Meanwhile, relative motion of polymers causes friction energy owing to the abundant H-bonds. This steady deformation leads to the stress plateau.

### 3.4. Effect of Density on Mechanical Properties

**Figure 10:**
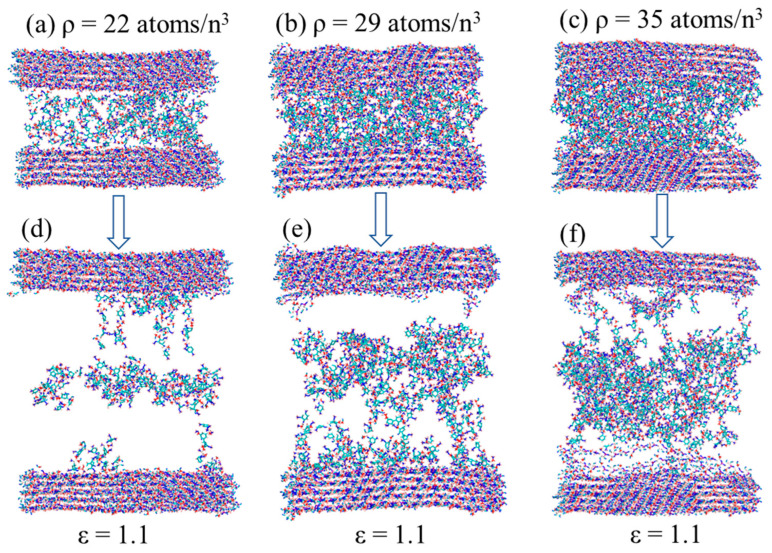
Atomic configurations of cellulose–lignin–cellulose with densities of (**a**) 22 atom/n^3^, (**b**) 29 atoms/n^3^, and (**c**) 35 atoms/n^3^ after relaxation. (**d**–**f**) are deformed configurations at the strain of 1.1.

To date, it is still ambiguous of how dense the cellulose, hemicellulose, and lignin are arranged in coconut endocarp. We hypothesize that the compact density of polymers may affect the deformation mechanism and hence influence the mechanical properties of entire system. In this subsection, take the C–L–C for instance, we generated three models with different density of 22 atoms/n^3^, 29 atoms/n^3^, and 35 atoms/n^3^, and relaxed, as displayed in Figure 10a–c. For simplicity, we named them as loose, moderate, and dense cases, respectively.

**Figure 11:**
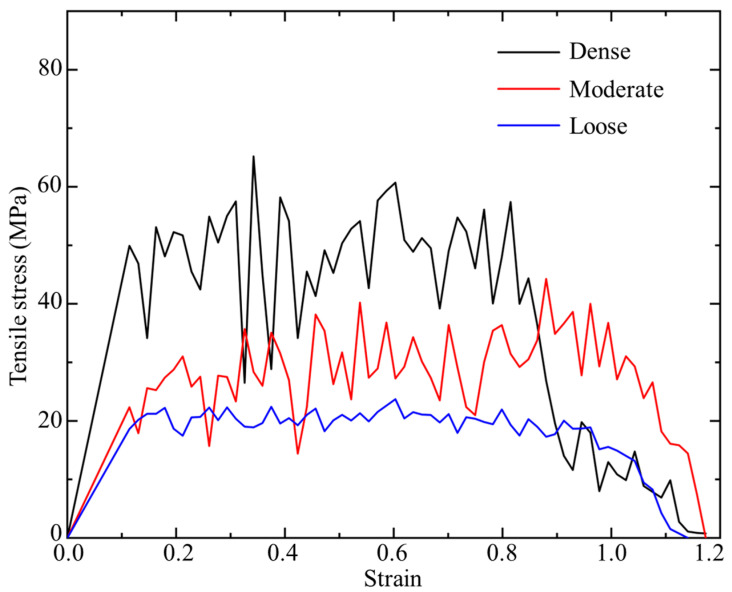
Stress–strain curves of cellulose–lignin–cellulose with different densities under uniaxial tension.

After uniaxial tension, the stress–strain curves are plotted in Figure 11. It can be seen that the stiffness, strength, and toughness increase with the increase in density. Additionally, one may note that a dramatic jump of stiffness and strength occurs from the moderate case to the dense case, which implies that a critical density may exist between 29 atoms/n^3^ and 35 atoms/n^3^. In the loose case, a relatively smooth stress–strain curve was observed. The steady but less vibrated stress plateau indicates that less disentanglement and friction of polymer chains are involved, as evidenced in Figure 10d. Lignin is deformed in a relatively uniform manner. For the moderate and dense cases, fluctuations of stress–strain curves in plastic region become severe. From Figure 10e,f, we notice that more lignin chains stick to the cellulose surfaces, which is primarily responsible for the high strengths obtained in Figure 11. Comparing to the uniformly stretched lignin chains in the moderate case (Figure 10e), an agglomerated lump was observed in the dense case (Figure 10f). These differences in deformation mode could not only elucidate the different stress–strain response, but also inspire ideas in materials design.

### 3.5. Effect of Number of Polymer Layers on Mechanical Properties

**Figure 12:**
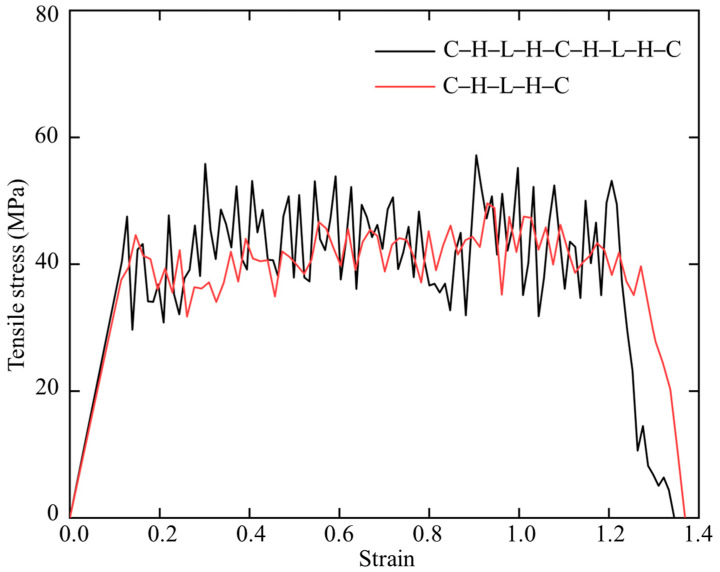
Stress–strain curves of nine layers cellulose–hemicellulose–lignincellulose–lignin–hemicellulose-cellulose–hemicellulose–lignincellulose–lignin–hemicellulose–cellulose (C–H–L–H–C–H–L–H–C) and five layers cellulose–hemicellulose–lignincellulose–lignin–hemicellulose–cellulose (C–H–L–H–C) under uniaxial tension.

**Figure 13:**
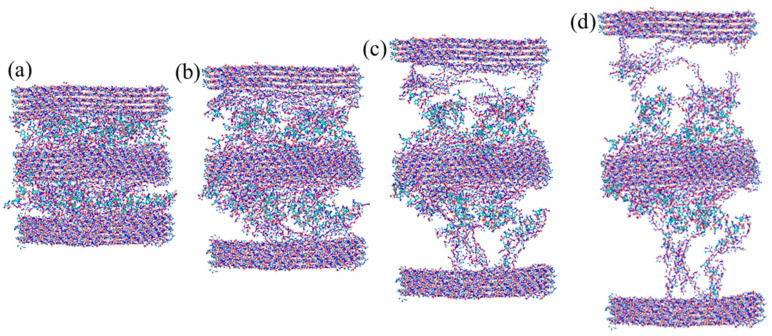
Snapshots of nine layers cellulose–hemicellulose–lignincellulose–lignin–hemicellulose-cellulose–hemicellulose–lignincellulose–lignin–hemicellulose–cellulose (C–H–L–H–C–H–L–H–C) (**a**) after relaxation, and under tensile strains of (**b**) 0.3, (**c**) 0.6, and (**d**) 1.1.

Facilitated by FIB microscope, multilayer polymers with lamellar thicknesses of 527 ± 105 nm and pores with 69 ± 19 nm in diameter were observed in the secondary cell wall of coconut endocarp at submicron scale [7]. However, the arrangement of polymers at nanoscale is still unclear. Given that endocarp exhibit a hierarchical structure and inspired by the layered cellulose–hemicellulose/lignin structure reported in wood, we hypothesize that cellulose, hemicellulose, and lignin may form multilayer in endocarp. Therefore, we tested the effect of number of polymer layers on the mechanical response. In the context, we built nine layers of C–H–L–H–C–H–L–H–C to perform the uniaxial tension test. The obtained stress–strain curve was compared with that of the five layers C–H–L–H–C in Figure 12. It can be seen that the nine layers model exhibit a slightly higher average strength than the five layers model. Figure 13 presents the deformed structures of nine layers C–H–L–H–C–H–L–H–C under tension. Compared to the five layers C–H–L–H–C in Figure 9b, we found that most of in-between amorphous polymers tend to attach to the middle cellulose bundle. A discontinuity between hemicellulose and lignin was observed, and it finally cause the failure of the entire system.

## 4. Conclusions

In this work, SMD, DFT, and classic MD simulations were performed to study the interaction among the cellulose, hemicellulose, and lignin in coconut endocarp. Polymer couples were generated and sheared through SMD simulations. Adsorption energies were quantified to validate the SMD results. Sandwiched multilayer polymer models were created to study the mechanical response and failure mechanisms under uniaxial tension. The effects of density and number of polymer layers on deformation behaviors were also investigated.

SMD shear simulation and DFT calculations confirmed that cellulose–hemicellulose and cellulose–lignin exhibit the strongest and weakest interaction, respectively, due to the different number of H-bonds form during the shear process. Uniaxial tension test of sandwiched polymer models revealed that C–H–C/C–H–L–H–C are stiffer, stronger and tougher than C–L–C/C–H–L–C, owing to the weak interface between cellulose–lignin. It is speculated that there exists a critical density that could lead to remarkable mechanical properties. As multiple cellulose bundles involved, the mechanical response varies.

Together, the fundamental knowledge gained in this work on the interaction between cellulose–hemicellulose–lignin provides an improved understanding of deformation mechanisms in coconut endocarp and other complex cellular materials, providing new hypotheses and design guidelines for the development of strong, ductile, and tough bioinspired materials. 

## Data Availability

The data that support the findings of this work are available from the corresponding author upon reasonable request.
